# A Diagnostic Pitfall in the Emergency Department —Aortic Dissection Masquerading as Acute Paraplegia: A Case Report

**DOI:** 10.5811/cpcem.61883

**Published:** 2026-08-03

**Authors:** Sindujaa Nagarajan, Ezhilkugan Ganessane, B G Kowsthubha, Anukarthika Somasundaram, Nathan Balamurugan

**Affiliations:** * Jawaharlal Institute of Postgraduate Medical Education and Research, Department of Emergency Medicine and Trauma, Puducherry, India; † All India Institute of Medical Sciences, Department of Trauma & Emergency, Madurai, India

**Keywords:** aortic dissection, acute paraplegia, anterior spinal artery syndrome, spinal cord ischemia, case report

## Abstract

**Introduction:**

Acute aortic dissection is a life-threatening emergency with a wide spectrum of clinical presentations. Neurological deficits as the initial manifestation of aortic dissection are uncommon and may result in delayed or missed diagnosis. This case describes aortic dissection presenting primarily as acute paraplegia without persistent chest pain or other classic features.

**Case Report:**

We report a 52-year-old male who presented with sudden-onset paraplegia. Neurological examination revealed flaccid paralysis of both lower limbs, with loss of pain and temperature sensation below the first lumbar dermatome and preserved dorsal column modalities. The patient was hypertensive on presentation. A clinical diagnosis of anterior spinal artery syndrome was made. Contrast-enhanced computed tomography of the chest and abdomen demonstrated a Stanford type A aortic dissection. The patient was counseled regarding the need for urgent surgical repair but declined operative intervention and opted for conservative management. His hospital course was complicated by progressive renal failure, and he subsequently succumbed to it.

**Conclusion:**

This case underscores the importance of considering aortic dissection in patients presenting with acute, nontraumatic paraplegia, even in the absence of persistent chest pain or other classic features.

## INTRODUCTION

Aortic dissection is a medical and surgical emergency associated with high morbidity and mortality. Classic symptoms include sudden-onset severe chest or back pain, pulse deficits, and hemodynamic instability.^1^ However, up to one-third of patients may present atypically, including neurological manifestations such as stroke, syncope, or paraplegia.^2^

Acute paraplegia usually prompts evaluation for spinal cord compression, including causes such as tumor, epidural abscess, hematomyelia, transverse myelitis, electrolyte abnormalities (eg, hypokalemia), or acute inflammatory demyelinating polyneuropathy. A vascular etiology, such as aortic dissection, has a wide spectrum of clinical presentations and may mimic other cardiovascular or neurological conditions. Hence, it can lead to delayed or missed diagnosis, particularly in the absence of classic symptoms such as persistent chest pain.^3^ Spinal cord ischemia in aortic dissection results from compromised perfusion of spinal arteries, most commonly the anterior spinal artery, leading to anterior spinal artery syndrome. We report a case of Stanford type A aortic dissection presenting primarily as acute paraplegia, emphasizing the need for heightened diagnostic suspicion in the emergency department (ED).

## CASE REPORT

A 52-year-old man was well until the morning of presentation, when he developed an episode of central, nonradiating chest pain associated with diaphoresis and palpitations. The pain lasted for a few minutes and resolved spontaneously, with no associated dyspnea or syncope. Shortly thereafter, he developed tingling and numbness in both lower limbs, which rapidly progressed to complete paralysis, accompanied by urinary incontinence. Approximately three to four hours after symptom onset, he was taken to a nearby hospital, where computed tomography of the lumbar spine was performed to evaluate for spinal pathology and showed no acute abnormality. As the neurological deficit persisted without a clear diagnosis, he was referred to our tertiary care center, where he arrived approximately 17 hours after symptom onset.

His medical history was notable only for congenital blindness. There was no known history of hypertension, diabetes mellitus, or connective tissue disease.

On arrival, the patient was alert and oriented. Vital signs were as follows: heart rate, 76 beats per minute; respiratory rate, 20 breaths per minute; blood pressure, 170/110 mm Hg measured in the right upper limb; temperature; 37 °C; and oxygen saturation, 98% on room air. The general physical examination was unremarkable. Cardiovascular examination demonstrated normal heart sounds without murmurs. Respiratory and abdominal examinations were normal.

Neurological examination revealed paralysis of both lower limbs with motor strength 0/5, absent deep tendon reflexes, and mute plantar responses. Sensory examination demonstrated loss of pain and temperature sensation below the first lumbar dermatome with preserved vibration and proprioception, consistent with anterior spinal artery syndrome (Figure). Cranial nerve and upper limb examinations were normal. Peripheral pulses were palpable and symmetric in all four extremities.

*CPC-EM Capsule*

What do we already know about this clinical entity?

*Aortic dissection is life-threatening and may present atypically with neurological deficits, including spinal cord ischemia causing paraplegia.*

What makes this presentation of disease reportable?

*Type A dissection presented as isolated acute paraplegia without persistent chest pain or pulse deficit, mimicking anterior spinal artery syndrome.*

What is the major learning point?

*Consider aortic dissection in acute nontraumatic paraplegia, even without chest pain, to avoid diagnostic delay and fatal outcomes.*

How might this improve emergency medicine practice?

*Broader differentials in paraplegia and early vascular image could reduce missed aortic dissections and improve survival.*

**Image 1:**
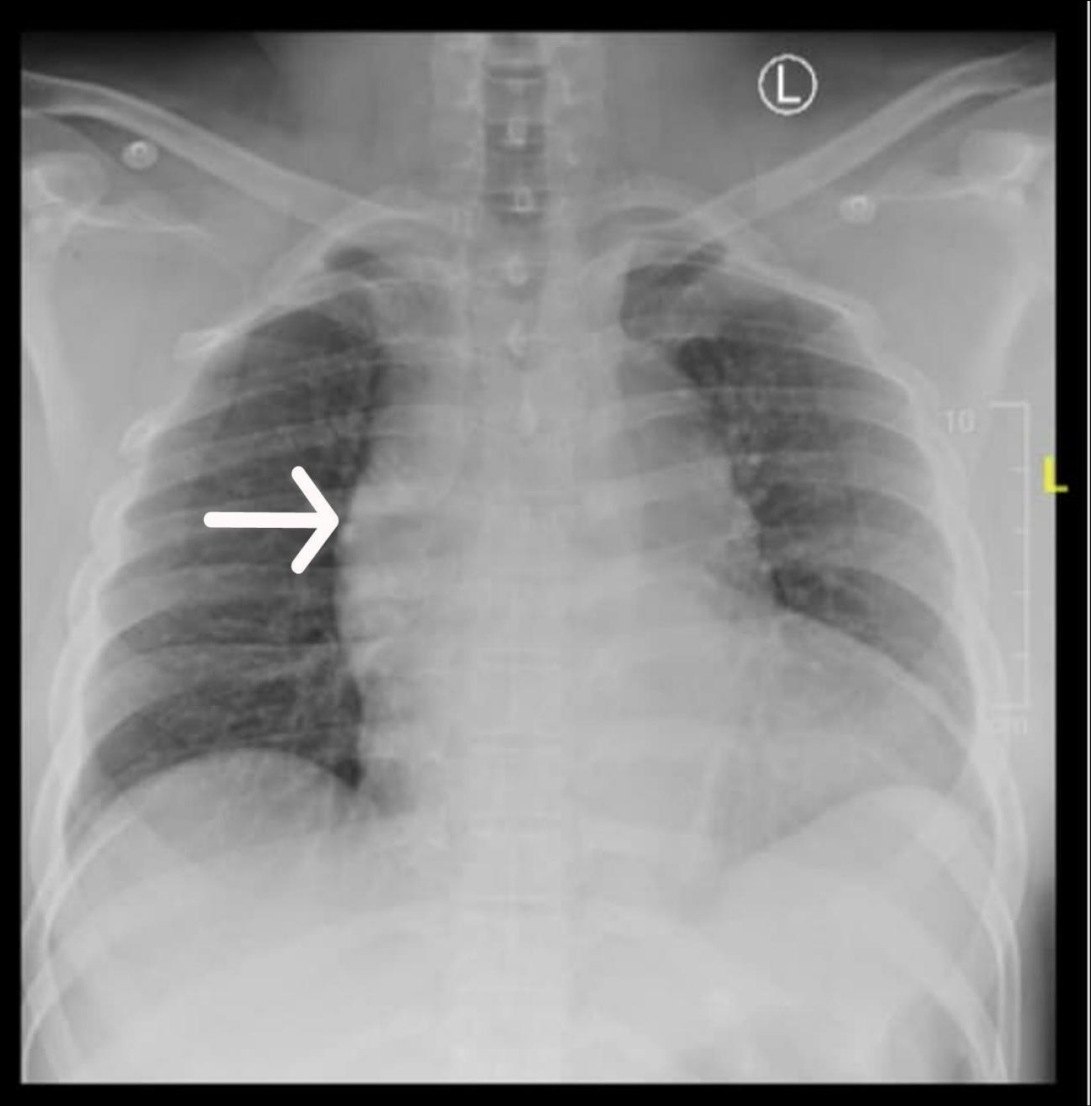
Chest radiograph demonstrating a widened mediastinum, a classic finding in aortic pathology.

Approximately six hours after arrival, while he was under observation in the ED, he developed worsening tachypnea, prompting arterial blood gas analysis. The arterial blood gas analysis revealed a high anion gap metabolic acidosis with elevated lactate levels, raising concern for systemic hypoperfusion. Based on the neurological findings and concomitant lactic acidosis, a vascular etiology was considered. A chest radiograph demonstrated a widened mediastinum (Image 1). Lab studies showed the following: hemoglobin,12.9 g/dL (reference range, 13.1–17.2 g/dL); urea, 40 mg/dL (17–43 mg/dL); and creatinine, 1.9 mg/dL (0.6–1.17 mg/dL).

**Image 2:**
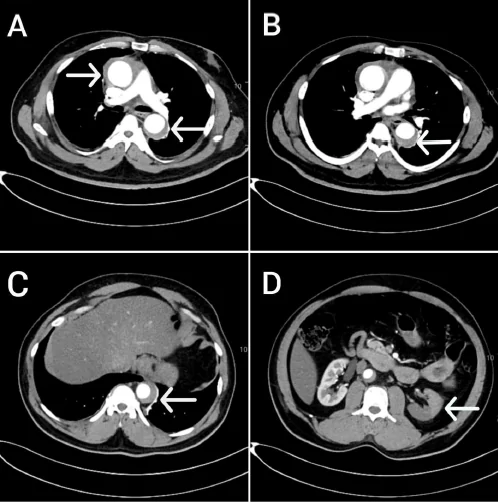
Computed tomography of the chest and abdomen demonstrating Stanford type A aortic dissection. Panel A: periadventitial hematoma surrounding the ascending aorta, extending to the aortic arch and descending thoracic aorta. Panel B: an intramural hematoma in the descending thoracic aorta. Panel C: the descending thoracic aorta with a partially thrombosed false lumen at the level of the thoracic spinal cord. Panel D: a nonopacified left kidney on contrast-enhanced imaging, as the left renal artery arose from the false lumen.

Because a vascular etiology, such as aortic dissection, was suspected, blood pressure was measured in all four limbs and noted to be 170/110 mm Hg in the right upper limb, 182/100 mm Hg in the left upper limb, and 180/100 mm Hg in both lower limbs. Contrast-enhanced computed tomography of the chest and abdomen was performed approximately eight hours after presentation. The delay in vascular imaging was due to an initial diagnostic dilemma and hospital-related logistical constraints. The imaging demonstrated a Stanford type A aortic dissection involving the ascending aorta and aortic arch, associated with aneurysmal dilatation, with extension into the descending thoracic aorta. The superior mesenteric and celiac arteries arose from the true lumen. The right renal artery originated from the true lumen, while the left renal artery arose from the false lumen. Partial thrombosis of the false lumen was noted, without evidence of active hemorrhage (Image 2).

The patient was counseled regarding the need for urgent surgical repair, but he declined operative intervention and opted for supportive medical management. Blood pressure was controlled with an intravenous labetalol infusion. Despite aggressive medical management, the patient developed progressive renal failure secondary to left renal artery ischemia and worsening metabolic acidosis. He underwent multiple sessions of hemodialysis but continued to deteriorate and subsequently succumbed five days after admission due to uremic complications.

## DISCUSSION

Aortic dissection is a relatively uncommon condition with an estimated incidence of 5–30 cases per one million individuals per year.^3^ Chest pain is not obligatory, and pain-free dissections have been reported in 5–15% of cases, particularly among patients with neurological manifestations.^4^,^5^ Neurological complications occur in 17–40 % of patients with aortic dissection, although paraplegia remains rare.^2^,^3^

Spinal cord ischemia results from compromised spinal cord perfusion due to arterial occlusion, hypotension, or thrombosis. Involvement of the anterior spinal artery produces anterior spinal artery syndrome, characterized by paraplegia, areflexia, loss of pain and temperature sensation, and preservation of vibration and proprioception, as observed in our patient.^6^

Similar cases of aortic dissection presenting with bilateral lower extremity weakness have been described in the emergency medicine literature. In a case report by Rao et al, the presentation was initially suspected to be traumatic in origin; however, early suspicion for aortic dissection arose due to absent distal pulses in both lower extremities.^7^ In a recent report by Campa et al, the patient presented with chest pain accompanied by bilateral lower limb weakness, with complete loss of all sensory modalities.^8^ In contrast, our patient demonstrated a neurological pattern consistent with anterior spinal artery syndrome, with preservation of vibration and proprioception but loss of pain and temperature sensation. This distinctive sensory pattern, along with the absence of pulse deficit, contributed to the initial diagnostic uncertainty and delayed consideration of aortic pathology.

In the ED, acute nontraumatic paraplegia often prompts evaluation for primary neurological causes, and vascular etiologies may be overlooked, particularly in the absence of persistent chest pain. This case illustrates diagnostic anchoring on a neurological syndrome, leading to delayed consideration of a vascular catastrophe. Emergency physicians should be cautious about attributing acute paraplegia solely to primary spinal cord pathology and should consider vascular etiologies such as aortic dissection when evaluating patients with acute neurological deficits. Although blood pressure measurements and assessment for pulse deficits in all four limbs were unremarkable, the subsequent development of worsening tachypnea with lactic acidosis and a widened mediastinum on chest radiography raised concern for an underlying vascular pathology. Pulse deficit is a classic physical examination finding in aortic dissection, but it is present in only 19–30% of cases and, therefore, cannot be relied upon to exclude the diagnosis.^9^ Emergency physicians should maintain a high index of suspicion for aortic dissection in patients presenting with acute paraplegia, particularly when accompanied by signs of end-organ hypoperfusion.

The blood supply of the spinal cord is complex. The artery of Adamkiewicz, the largest radicular artery, supplies the lower thoracic and lumbar spinal cord and typically arises between the ninth thoracic and second lumbar, most often from the left side of the aorta. Its variable anatomy predisposes it to ischemia during aortic pathology, including dissection.^10^

Hypertension is the most prevalent risk factor for aortic dissection, reported in up to 75% of cases, and it was also present in our patient.^11^ However, despite its strong association as a risk factor, the presence of hypertension has low specificity and limited accuracy for diagnosing aortic dissection, as it is frequently encountered in other acute conditions presenting with neurological deficits.^12^ Initial medical management with adequate pain control and blood pressure reduction is essential in all patients. Beta blockers are first-line agents due to their ability to reduce shear stress on the aortic wall. Vasodilators may be added after beta-blockade to achieve a target systolic blood pressure, generally between 100–120 mm Hg, in the absence of aortic regurgitation.^13^

Definitive management of Stanford type A aortic dissection is urgent surgical repair, with mortality approaching 50% within 48 hours without intervention.^13^ Thoracic endovascular aortic repair has emerged as an option for complicated type B dissections.^13^ The reported mortality associated with spinal cord infarction ranges from 9–23%, underscoring the poor prognosis associated with this complication.^14^,^15^

## CONCLUSION

Aortic dissection may present solely as acute paraplegia without persistent chest pain. Although rare, this presentation carries a high risk of mortality if unrecognized. Emergency physicians should maintain a broad differential diagnosis when evaluating nontraumatic paraplegia and consider early contrast imaging when clinical suspicion for aortic pathology exists.

## Figures and Tables

**Figure:**
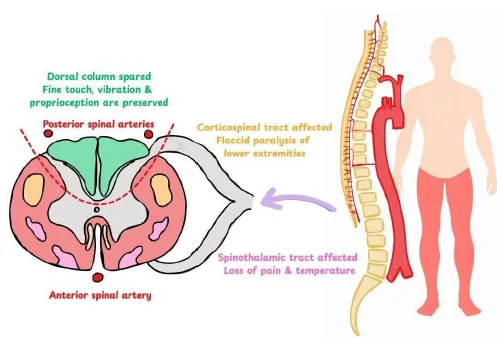
Diagrammatic representation of anterior spinal artery syndrome demonstrating loss of motor function and pain and temperature sensation with preservation of dorsal column modalities.
